# Ultra-Sensitive Detection of *Plasmodium falciparum* by Amplification of Multi-Copy Subtelomeric Targets

**DOI:** 10.1371/journal.pmed.1001788

**Published:** 2015-03-03

**Authors:** Natalie Hofmann, Felista Mwingira, Seif Shekalaghe, Leanne J. Robinson, Ivo Mueller, Ingrid Felger

**Affiliations:** 1 Swiss Tropical and Public Health Institute, Basel, Switzerland; 2 University of Basel, Basel, Switzerland; 3 Biological Sciences Department, Dar es Salaam University College of Education, Dar es Salaam, Tanzania; 4 Ifakara Health Institute, Bagamoyo, Tanzania; 5 Papua New Guinea Institute of Medical Research, Madang and Maprik, Papua New Guinea; 6 Walter and Eliza Hall Institute, Parkville, Victoria, Australia; 7 Department of Medical Biology, University of Melbourne, Parkville, Victoria, Australia; 8 Centre de Recerca en Salut Internacional de Barcelona, Barcelona, Spain; Mahidol-Oxford Tropical Medicine Research Unit, THAILAND

## Abstract

**Background:**

Planning and evaluating malaria control strategies relies on accurate definition of parasite prevalence in the population. A large proportion of asymptomatic parasite infections can only be identified by surveillance with molecular methods, yet these infections also contribute to onward transmission to mosquitoes. The sensitivity of molecular detection by PCR is limited by the abundance of the target sequence in a DNA sample; thus, detection becomes imperfect at low densities. We aimed to increase PCR diagnostic sensitivity by targeting multi-copy genomic sequences for reliable detection of low-density infections, and investigated the impact of these PCR assays on community prevalence data.

**Methods and Findings:**

Two quantitative PCR (qPCR) assays were developed for ultra-sensitive detection of *Plasmodium falciparum*, targeting the high-copy telomere-associated repetitive element 2 (TARE-2, ∼250 copies/genome) and the *var* gene acidic terminal sequence (*var*ATS, 59 copies/genome). Our assays reached a limit of detection of 0.03 to 0.15 parasites/μl blood and were 10× more sensitive than standard 18S rRNA qPCR. In a population cross-sectional study in Tanzania, 295/498 samples tested positive using ultra-sensitive assays. Light microscopy missed 169 infections (57%). 18S rRNA qPCR failed to identify 48 infections (16%), of which 40% carried gametocytes detected by *pfs25* quantitative reverse-transcription PCR. To judge the suitability of the TARE-2 and *var*ATS assays for high-throughput screens, their performance was tested on sample pools. Both ultra-sensitive assays correctly detected all pools containing one low-density *P. falciparum*–positive sample, which went undetected by 18S rRNA qPCR, among nine negatives. TARE-2 and *var*ATS qPCRs improve estimates of prevalence rates, yet other infections might still remain undetected when absent in the limited blood volume sampled.

**Conclusions:**

Measured malaria prevalence in communities is largely determined by the sensitivity of the diagnostic tool used. Even when applying standard molecular diagnostics, prevalence in our study population was underestimated by 8% compared to the new assays. Our findings highlight the need for highly sensitive tools such as TARE-2 and *var*ATS qPCR in community surveillance and for monitoring interventions to better describe malaria epidemiology and inform malaria elimination efforts.

## Abstract 2

Ingrid Felger and colleagues developed an assay that targets multi-copy genomic sequences and can detect low-density infections with falciparum malaria parasites.

## Editors' Summary

**Background:**

Nearly half the world's population is at risk of malaria, and more than 600,000 people die from this mosquito-borne parasitic infection every year. Most of these deaths are caused by *Plasmodium falciparum*, which is transmitted to people by night-flying *Anopheles* mosquitoes. These insects inject “sporozoites” into people, a parasitic form that replicates inside human liver cells. After a few days, the liver cells release “merozoites,” which invade red blood cells, where they replicate rapidly before bursting out and infecting more red blood cells. This increase in parasitic burden causes malaria's characteristic fever, which needs to be treated promptly to prevent anemia and organ damage. Infected red blood cells also release “gametocytes,” which infect mosquitoes when they take a blood meal. In the mosquito, the gametocytes multiply and develop into sporozoites, thus completing the parasite's life cycle. Malaria can be prevented by controlling the mosquitoes that spread the parasite and by avoiding mosquito bites. Effective treatment with antimalarial drugs also helps to reduce malaria transmission and is a key component of global efforts to control and eliminate malaria.

**Why Was This Study Done?:**

Planning and evaluating malaria control and elimination efforts relies on having accurate and sensitive methods to measure parasite prevalence—the proportion of a population infected with parasites. It is particularly important to know how many people are carrying low-density infections because although these individuals have no symptoms, they contribute to malaria transmission. In the past, malaria was usually diagnosed by looking for parasites in blood using light microscopy, but molecular tests based on “quantitative polymerase chain reactions” (qPCRs) are now available that detect much lower parasite densities in blood (submicroscopic infections). qPCRs detect parasite-specific DNA sequences in patient blood samples, but reliable detection of low-density infections remains imperfect because the abundance of target sequences in patient samples limits the sensitivity of current qPCR methods. Here, the researchers investigate whether the sensitivity of *P. falciparum* detection using qPCR can be improved by targeting multi-copy genomic sequences—DNA sequences that are repeated many times in the parasite's genetic blueprint.

**What Did the Researchers Do and Find?:**

The researchers developed two new qPCRs for *P. falciparum* by using the telomere-associated repetitive element 2 (TARE-2; 250 copies/genome) and the *var* gene acidic terminal sequence (*var*ATS; 59 copies/genome) as target sequences. Direct comparison of these qPCRs with the standard 18S rRNA qPCR for *P. falciparum*, which targets a gene present at 5–8 copies/genome, indicated that the new assays were ten times more sensitive than the standard assay and could detect as few as 0.03–0.15 parasites/μl blood. Next, the researchers used light microscopy, 18S rRNA qPCR, and the two new qPCRs to look for *P. falciparum* parasites in 498 samples randomly selected from a malaria survey undertaken in Tanzania. Parasite prevalences were 25% by light microscopy, 50% by 18S rRNA qPCR, and 58% by TARE-2 or varATS qPCR. Compared to TARE-2 or *var*ATS qPCR, 18S rRNA qPCR failed to identify 48 infections (16% of infections). Moreover, 40% of the positive samples missed by 18S rRNA qPCR contained gametocytes (detected by a different PCR-based assay) and therefore came from individuals capable of transmitting malaria parasites to mosquitoes. Finally, to test the suitability of the new ultra-sensitive assays for use in high-throughput screens, the researchers tested performance of the assays on sample pools. Both tests correctly identified all pools containing one low-density *P. falciparum*–positive sample among nine negative samples, whereas 18S rRNA qPCR identified none of these pools.

**What Do These Findings Mean?:**

These findings provide evidence of low-density malaria infections in individuals previously thought to be parasite-free, even after testing with a molecular diagnostic. Notably, in the population considered in this study, the standard 18S rRNA qPCR underestimated parasite prevalence by nearly 10%. The assays developed in this study have some important limitations, however. First, they detect only *P. falciparum*, and malaria control programs ideally need assays that detect all the *Plasmodium* species that cause malaria. Second, because the TARE-2 and *var*ATS qPCRs require advanced laboratory infrastructure, they cannot be used in remote field settings. Nevertheless, because low-density infections are likely to become increasingly common as countries improve malaria control, these findings highlight the need for ultra-sensitive tools such as the TARE-2 and *var*ATS qPCRs for community surveillance and for monitoring the progress of malaria control and elimination programs.

**Additional Information:**

Please access these websites via the online version of this summary at http://dx.doi.org/10.1371/journal.pmed.1001788.

- Information is available from the World Health Organization on malaria (in several languages), including information on malaria diagnosis; the *World Malaria Report 2014* provides details of the current global malaria situation
- The US Centers for Disease Control and Prevention also provides information on all aspects of malaria; its website provides a selection of personal stories about malaria
- Information is available from the Roll Back Malaria Partnership on the global control of malaria and on the Global Malaria Action Plan (in English and French)
- MedlinePlus provides links to additional information on malaria (in English and Spanish)

## Introduction

Accurate and sensitive detection of malaria parasites is a key factor in planning, targeting, and evaluating malaria control efforts, and requires different strategies at different elimination stages [1–3]. One major challenge is the identification of remaining reservoirs of human-to-mosquito transmission in asymptomatic individuals carrying low-density infections. The true extent of this predominantly submicroscopic reservoir became better defined with the wider application of molecular detection techniques in epidemiological studies [4,5], and its relevance to sustained malaria control has been brought into focus [1–3]. It was recently estimated that submicroscopic but PCR-detectable infections make up 20% of all malaria infections in high-transmission areas and as much as 70% in low-endemic areas, where they contribute 40% of all transmission to mosquitoes [5]. Mass drug administration (MDA) interventions include treatment of these undetected carriers and can thereby reduce parasite prevalence for several months in low- to moderate-prevalence settings, with even longer effects predicted at low transmission levels [6,7]. According to modeling predictions, mass screening and treatment (MSAT) strategies have a lower impact than MDA-based interventions [7], as MSAT is limited by the sensitivity of the diagnostic tool used. A recent study in Burkina Faso found no sustained effect of anti-malarial treatment on incidence of clinical episodes 9 mo after MSAT using conventional diagnosis based on rapid diagnostic test (RDT) [8]. This finding is likely attributable to the large proportion of undetected low-density infections. The true parasite burden could be better defined using nucleic-acid-based diagnostics, but even then, very-low-density infections might be missed. Such low-density infections might be particularly prevalent in areas with a recent and drastic decline in the force of infection of *Plasmodium falciparum*, where high parasite densities and disease are controlled by residual immunity. As more countries successfully reduce malaria prevalence [9], the proportion of low-density infections can be expected to rise, and more sensitive diagnostics that surpass even conventional PCR are urgently needed to detect potential hidden reservoirs.

Of the current molecular detection methods available for malaria diagnosis (summarized in Table 1), RNA-based techniques such as quantitative reverse transcription PCR (qRT-PCR) [10–12], nucleic acid sequence-based amplification (NASBA) [13–15], and ELISA-like hybridization assays [16] reach the highest sensitivities by targeting the highly abundant 18S small subunit ribosomal RNA (18S rRNA). However, because of the unstable nature of RNA, these assays require dedicated and controlled sample collection and storage, and thus have only a limited application in field settings. DNA-based techniques are generally more field-adaptable and include nested PCR [17–22], quantitative PCR (qPCR) [23–31], loop-mediated isothermal amplification (LAMP) [32–35], isothermal recombinase polymerase amplification (RPA) [36], and alternative PCR-based detection methods [19,37–41]. Of the DNA-based assays, only qPCR allows one to robustly quantify copy numbers of the template DNA in the reaction as a measure of parasite load in the sample.

**Table 1:** Assay characteristics and limit of detection (LOD) of published *P*. *falciparum* detection assays.

Method	Template Molecule	Target Gene	Quantification	LOD (Parasites/μl Blood)	Reference
Nested PCR	DNA	18S rRNA, *dhfr-ts*, 28S rRNA, *stevor*	No	0.1–10	[17–22]
PCR	DNA	mitochondrial DNA	No	0.5	[42]
qPCR	DNA	18S rRNA, *cox1*, *cytb*	Yes	0.02–3	[23–31]
PCR-based	DNA	18S rRNA, *cox1*	Yes/No	0.5–1	[19,37–41]
LAMP^a^	DNA	18S rRNA, mitochondrial DNA	No	1–10	[32–35]
RPA^a^	DNA	18S rRNA	No	4	[36]
qRT-PCR	RNA	18S rRNA	Yes	0.002–0.02	[10–12]
(QT-)NASBA^a^	RNA	18S rRNA	Yes/No	0.02	[13–15]

^a^Isothermal amplification process.

QT-NASBA, quantitative NASBA.

Due to the lower number of target molecules in the sample, DNA-based techniques have a reduced sensitivity compared to their RNA-based counterparts, but sampling for DNA-based diagnosis is more robust. The most prominent molecular marker is the 18S rRNA gene, present at 5–8 copies per genome, depending on the parasite strain [43]. In recent years, several attempts have been made to increase DNA-based PCR sensitivity by sampling larger blood volumes and concentrating the DNA [44], or choosing mitochondrial [19,27,32,42] or nuclear multi-copy PCR targets [40,45]. Already in 1997, Cheng et al. designed a nested PCR that detected the conserved region of the subtelomeric *stevor* gene group, with many copies per genome [46], which had improved sensitivity over single-copy PCRs [47].

We have taken this approach further and have chosen high-copy subtelomeric sequences with the widest possible chromosomal distribution to develop novel qPCR assays for highly sensitive detection and quantification of *P*. *falciparum* in low-density infections. The telomere-associated repetitive element 2 (TARE-2) is a 1.6-kb-long block consisting of ten to twelve 135-bp repeat units with slightly degenerate sequences, interspersed by two 21-bp sequences [48,49]. The TARE-2 repeat is present at 24 of 28 subtelomeres in the *3D7* culture strain [49], which amounts to approximately 250–280 copies per genome, and is specific to *P*. *falciparum* strains [48].

The *var* gene family is located primarily in the subtelomere and was chosen to develop a second qPCR with a multi-copy target. The genome of the *3D7* culture strain harbors 59 *var* genes [49], and an estimated 50–150 copies are present in other parasite lines [50,51]. *var* genes encode the *P*. *falciparum* erythrocyte membrane protein 1 (*Pf*EMP1) and possess a transmembrane domain and one intron, with exons 1 and 2 encoding the extra- and intracellular parts of *Pf*EMP1. In contrast to the highly variable extracellular domain, the intracellular *var* gene acidic terminal sequence (*var*ATS) comprises some well-conserved stretches and can thus be targeted by qPCR [50,51].

With the aim of increasing test sensitivity at least 10-fold and improving the robustness of parasite detection at low densities, we developed two novel qPCR assays using the multi-copy TARE-2 and *var*ATS sequences as targets. We then investigated the potential of both assays to detect ultra-low-density infections that are beyond the detection limit even of 18S rRNA qPCR. We further hypothesized that the abundance of the PCR target in the parasite genome would counterbalance the diluting effect of sample pooling, and thus tested the suitability of our assays for application to sample pools.

## Methods

### Ethical Approval

Field samples used for these analyses were derived from a cohort study conducted in Maprik District, Papua New Guinea (PNG), from 17 August 2009 to 20 May 2010 [11] and a cross-sectional survey conducted in Rufiji, Tanzania (TZ), in 2013. Scientific approval and ethical clearance for the PNG cohort study was obtained from the Medical Research and Advisory Committee of the Ministry of Health in PNG (MRAC no. 09.24) and the Ethics Commission of Basel Land and Basel Stadt (no. 237/11). Approval for the TZ cross-sectional study was obtained from the Institutional Review Board of the Ifakara Health Institute, Dar es Salaam, TZ (no. 13-2013). Informed consent was obtained from all study participants in PNG and TZ, for children from parents or legal guardians prior to sampling.

### Primer Design and qPCR Conditions

For *var*ATS primer design, all 59 *var*ATS sequences per *P*. *falciparum* genome (strain *3D7*; PlasmoDB) were aligned using ClustalW2 (http://www.ebi.ac.uk/Tools/msa/clustalw2/). Within the size-polymorphic *var*ATS domain (size range 1–1.5 kb), the most conserved domain was selected for primer and MGB (minor groove binder) probe design. One wobble each was inserted into the forward primer and probe to improve annealing, whereas the reverse *var*ATS primer matched very well with all *3D7 var*ATS sequences. We expect that only about 40% of *3D7* sequences match sufficiently well with the selected oligonucleotides to yield an amplification product. Attempts to further increase assay sensitivity by using additional wobbles and combinations of primers were not successful. Primer and probe sequences, as well as qPCR mixes and cycling conditions, are listed in S1 Table.

The TARE-2 repeat region was identified in the genome of *P*. *falciparum* strains *3D7* (National Center for Biotechnology Information) and *IT* (PlasmoDB) using the Tandem Repeats Finder tool (http://tandem.bu.edu/trf/trf.html). TARE-2 sequences of other *P*. *falciparum* strains were retrieved by BLAST (http://blast.ncbi.nlm.nih.gov) search using *3D7* and *IT* repeat units. All repeat units were aligned using Clustal Omega (http://www.ebi.ac.uk/Tools/msa/clustalo/), and primers were designed on the most conserved stretches so that eight nucleotides prior to the 3′ end matched with the majority of repeat sequences. One wobble was inserted into each primer for better annealing. Owing to repeat degeneration and therefore difficult probe design, probe-free SYBR Green–based real-time quantification of amplicons was chosen. Primer sequences and qPCR reaction and cycling conditions are specified in S1 Table. The melt curves of amplicons were inspected in each experiment to detect false positivity. True positive samples differed clearly from primer dimer and unspecific PCR products based on the amplicon’s melting temperature (*T*_m_; S1 Fig).

Samples were quantified using a standard curve of plasmid (*var*ATS) or parasite genomic DNA (gDNA; TARE-2). As *var*ATS standard, the *var*ATS amplicon was amplified from *3D7* gDNA and inserted into the TOPO TA vector (Invitrogen). The purified plasmid was diluted to 10^6^, 10^4^, and 10^2^ plasmids/μl in TE buffer. As TARE-2 standard, gDNA of a 10-fold dilution of ring-stage *3D7* parasite culture was used (6.8 × 10^3^ to 6.8 × 10^−2^ parasites/μl; described in S1 Text).

The reference 18S rRNA qPCR was performed as described previously [11,23], using a MGB probe (6FAM-5′-ACGGGTAGTCATGATTGAGTT-3′-NFQ-MGB) in a total volume of 12 μl. DNA volume matched that of *var*ATS and TARE-2 qPCRs. The amount of target DNA in each sample was calculated from the *C*_*t*_ value using a plasmid standard curve as described above (18S rRNA amplicon inserted in TOPO TA vector [Invitrogen]). *pfs25* qRT-PCR for gametocyte detection was performed as described previously [11].

### Analytical Specificity and Sensitivity

The analytical specificity of the TARE-2 and *var*ATS qPCRs was assessed both in silico using BLAST search and experimentally using human gDNA from a healthy, malaria-free volunteer and *P*. *malariae* and *P*. *ovale* gDNA (three archived anonymized clinical patient samples each). No amplification from non-falciparum *Plasmodium* or human DNA was observed using the *var*ATS and TARE-2 qPCRs.

For assessment of *P*. *vivax* cross-reactivity, 14 samples with a low to medium number of genomic *P*. *vivax* 18S rRNA copies (22–393 *Pv*18S rRNA copies/μl; light microscopy [LM]: 0–219 parasites/μl) were selected from a previously analyzed sample pool [11]. All 14 selected *P*. *vivax* DNA samples had been diagnosed *P*. *falciparum*–negative by A18S qRT-PCR. All 14 samples were *var*ATS- and TARE-2-negative.

Analytical sensitivity and qPCR efficiency were validated on dilution rows of (i) in vitro cultured ring stages (*3D7* strain) and (ii) the WHO international standard for *P*. *falciparum* DNA nucleic acid amplification techniques (*National Institute for Biological Standards and Control*, UK) [27,52]. Details on generation of the dilution rows are presented in S1 Text. TARE-2 and *var*ATS qPCR efficiencies, determined on the *3D7* culture dilution row, were comparable to that of 18S rRNA qPCR; however, all qPCR efficiencies were slightly outside the desirable efficiency range of 90%–105% (Table 2). Efforts to optimize qPCR efficiency by varying primer concentration, annealing temperature, and qPCR volume were not successful.

**Table 2:** qPCR details and efficiencies of the 18S rRNA, *var*ATS, and TARE-2 assays.

Assay	Slope	Efficiency	Intercept^a^	*R* ^2^	Platform	Amplicon Length^b^	Amplified Copy Numbers in Genome
18S rRNA	−3.63	88.5%	41.09	1.0	TaqMan	221 bp	3
*var*ATS	−3.63	88.6%	34.50	1.0	TaqMan	65 bp	<59^c^
TARE-2	−3.75	84.7%	32.08	0.97	SYBR Green	93 bp	<250–280^c^

^a^Intercept equals the *C*_*t*_ value of the DNA equivalent of five parasites added to the qPCR reaction.

^b^Length of consensus sequence.

^c^Polymorphism in primer binding sites likely does not permit efficient amplification of all genomic copies. Number of target sequences present in parasite genomes from field samples cannot be determined in absence of the respective genome data.

### Field Samples and Nucleic Acid Extraction

In a pilot study, 60 DNA samples from PNG were used for assay validation. They were selected from a larger pool of previously analyzed samples based on their positivity in 18S rRNA qPCR (33 positives, 27 negatives), and we used 18S rRNA copy numbers in these samples to select a wide range of parasite densities [11]. DNA of PNG samples was extracted using the FavorPrep 96-well Genomic DNA Extraction Kit (Favorgen) from blood cell fractions of 50–150 μl, eluted in 200 μl of elution buffer, and stored at −20°C.

The 498 TZ samples were age-stratified randomly selected from the larger cross-sectional sample set, so that each age category contained at least 40 samples. We intended to estimate the overall proportion of *P*. *falciparum–*positive individuals by each test with a precision given by a CI of ±5%. Samples were collected as 50 μl of whole blood in 250 μl of RNAprotect Cell Reagent (Qiagen) to stabilize RNA. RNA extraction was performed as previously described [11]. DNA was co-extracted during RNA extraction using the RNeasy Plus 96 Kit (Qiagen). DNA was recovered from the gDNA eliminator column after two washing steps according to the QIAamp 96 DNA Blood Kit protocol (500 μl of AW1 buffer, 500 μl of AW2 buffer) and eluted in 100 μl of AE elution buffer.

TARE-2, *var*ATS, and 18S rRNA qPCR were performed once on each TZ DNA sample. If sample positivity did not agree between the three qPCR assays, each qPCR was repeated in duplicate for the discrepant sample, yielding a total of three qPCR replicates for all assays in the discrepant samples. Samples were defined as positive for *var*ATS, TARE-2, or 18S rRNA qPCR if two out of three replicates were positive. For gametocyte detection, *pfs25* qRT-PCR was performed as previously described using 4 μl of RNA [11].

### Generation of Pooled Samples

Low-density *P*. *falciparum*–positive samples (<2 parasites/μl by TARE-2 qPCR, LM negative) were selected from the TZ collection and mixed with four or nine *P*. *falciparum*–negative blood samples to create pools of five or ten samples. Negative samples were prepared by mixing 50 μl of blood from a malaria-negative blood donor with 250 μl of RNAprotect Cell Reagent (Qiagen) to permit simultaneous DNA and RNA isolation. Per sample, 100 μl of whole blood in RNAprotect Cell Reagent was added to the pool, resulting in a total sample volume of 500 μl or 1 ml (for five- and ten-sample pools, respectively). DNA was extracted from the entire volume of these pools using the RNeasy Plus 96 Kit (Qiagen) as described above, and DNA was eluted in 100 μl (five-sample pools) or 200 μl (ten-sample pools). In total we generated 20 pools of five samples, five of which contained a *P*. *falciparum*–positive sample, and ten pools of ten samples, two of which contained a positive sample.

### Statistical Analyses

Data analysis was performed using R v3.0.2 statistical software. The Mann-Whitney-Wilcoxon test was used to compare for each parasite population (TZ and PNG) the mean *T*_m_ of the specific amplicon versus primer dimer. The LOD of qPCR assays, i.e., the concentration at which a sample is detected with 95% confidence, was calculated at using probit analysis of the dilution row results. Proportions of samples positive for the TARE-2, *var*ATS, and 18S rRNA qPCRs in the TZ and PNG datasets were compared using McNemar’s Chi^2^ test. Correlations of parasite quantity per microliter or template copy number per microliter between assays were calculated using Pearson’s product-moment correlation.

## Results

### Limit of Detection of *var*ATS and TARE-2 qPCRs

**Fig 1:**
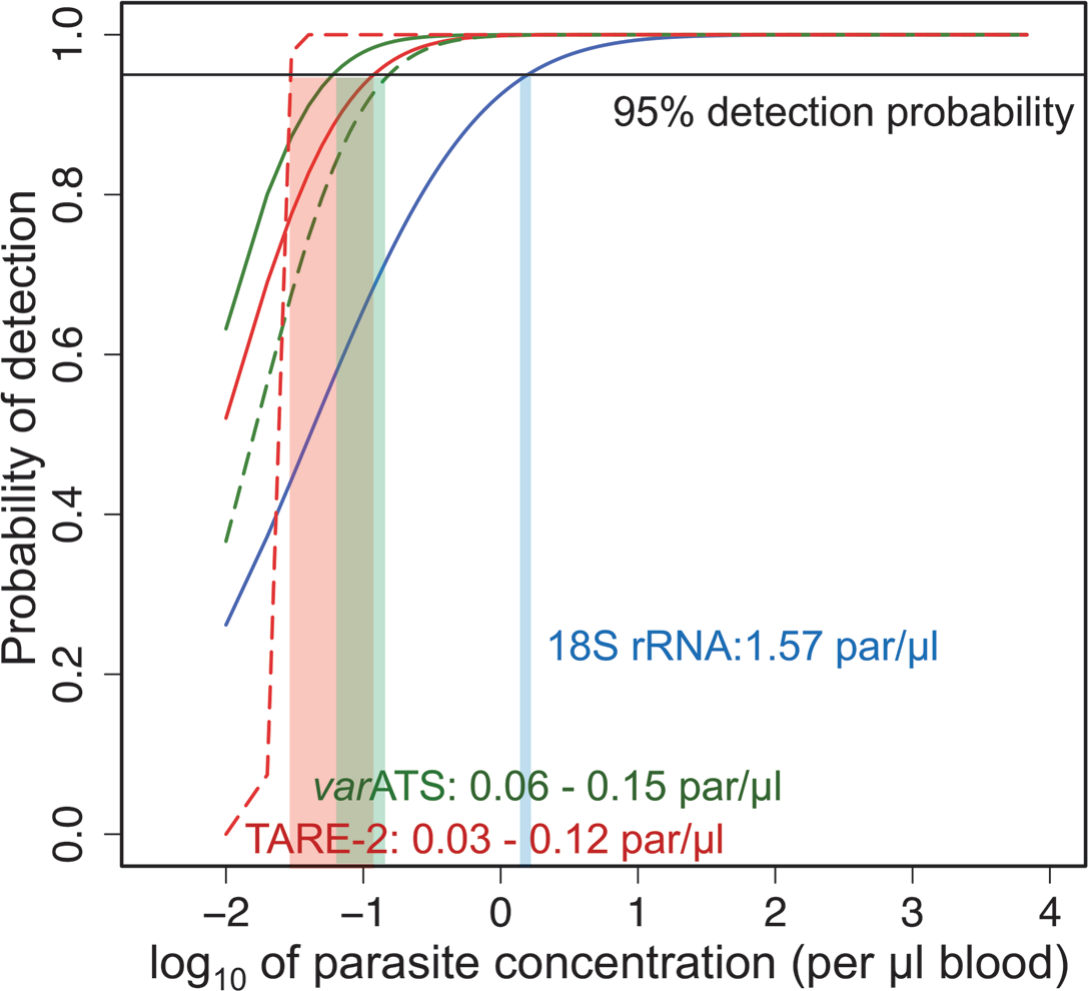
Limit of detection of TARE-2, *var*ATS, and 18S rRNA qPCRs. Dashed lines: based of serial dilution of WHO standard material [52]. Continuous lines: based on serial dilution of ring-stage *3D7* in vitro culture. par, parasites.

Probit analysis was used to determine the LOD, i.e., the concentration at which a sample would be detected with 95% confidence (Fig 1), based on qPCR results from dilution rows of parasite culture and WHO standard material (S3 Table). The *var*ATS and TARE-2 qPCRs were at least 10× more sensitive than standard 18S rRNA qPCR and reached LODs, calculated on the two different dilution rows, of 0.06 and 0.15 parasites/μl (*var*ATS; CI_95_ [0.02–1.07] and [0.05–4.37]) and 0.03 and 0.12 parasites/μl (TARE-2; CI_95_ [not defined] and [0.04–2.06]). Probit analysis of the TARE-2 results using the WHO standard dilution row did not yield a 95% CI because of the steep slope of the regression line. The LOD of 18S rRNA qPCR was calculated at 1.57 parasites/μl (CI_95_ [0.28–626.73]). The TARE-2 and *var*ATS assays can therefore robustly detect as few as 6–24 and 12–30 parasites in 200 μl whole blood, respectively, which is the typical volume normally processed for DNA extraction from fingerprick blood samples without sample concentration.

### Detection of Ultra-Low-Density Infections in Maprik District, Papua New Guinea

As pilot study, we compared the ability of the three qPCRs to detect low-density *P*. *falciparum* infections in 60 DNA samples from PNG. All 33 samples that were positive in 18S rRNA qPCR were also positive using both ultra-sensitive assays. Out of the 27 samples negative by 18S rRNA qPCR, four were positive in *var*ATS qPCR (McNemar’s Chi^2^, *p* = 0.181). The same four samples plus five additional samples were positive by TARE-2 qPCR, resulting in a significant gain in sample positivity (McNemar’s Chi^2^, *p* = 0.036). Since samples were not randomly selected but chosen deliberately to include a wide parasite density range, this result does not reflect the true *P*. *falciparum* prevalence in Maprik District, PNG. Nevertheless the number of additional samples positive for *P*. *falciparum* demonstrates that a considerable proportion of infections may persist at ultra-low densities and remain undetected by standard qPCR.

### Prevalence of Ultra-Low-Density Infections and Gametocyte Carriage in Rufiji, Tanzania

**Fig 2:**
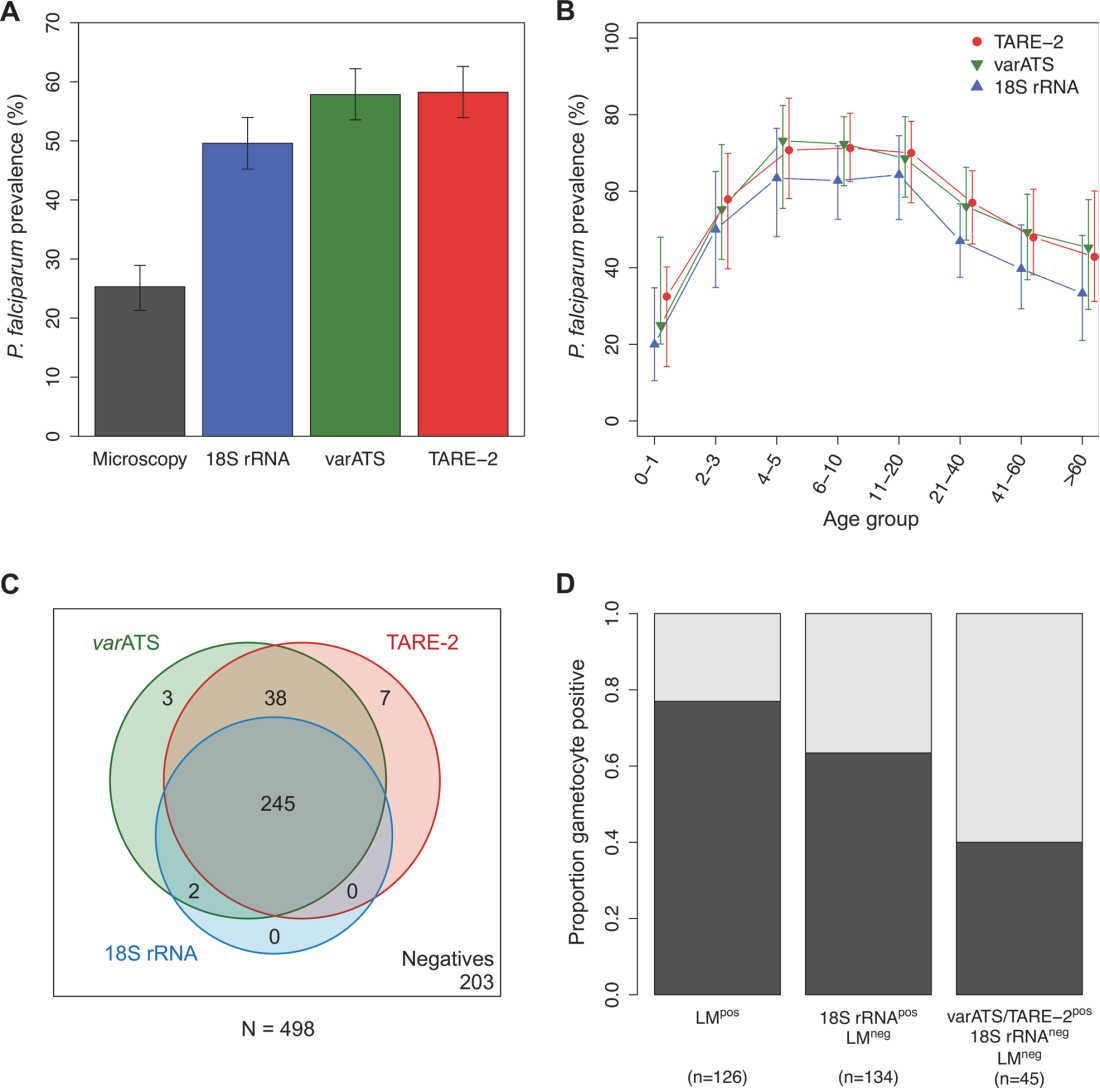
*P*. *falciparum* prevalence and gametocyte carriage in Rufiji, Tanzania. (A) Overall *P*. *falciparum* prevalence by different diagnostic methods. Error bars represent 95% CIs. (B) *P*. *falciparum* prevalence based on TARE-2, *var*ATS, and 18S rRNA qPCRs by age (in years). Error bars represent 95% CIs. (C) Venn diagram of positivity by *var*ATS, TARE-2, and 18S rRNA qPCRs. (D) Proportion of gametocyte carriers by *pfs25* qRT-PCR. Samples were categorized according to the least sensitive method that identified them as *P*. *falciparum*–positive. In total, 13 of 126 LM-positive samples were not confirmed by any qPCR, and 11 of these also were negative by RDT (SD Bioline Pan pLDH/PfHRP2), thus these samples should be considered false positive by LM. Three samples had to be excluded from the gametocyte analyses because of missing RNA data.

*P*. *falciparum* prevalence in Rufiji, TZ, was assessed in 498 samples randomly selected from a larger cross-sectional study conducted in 2013. *P*. *falciparum* prevalence was higher using ultra-sensitive detection methods as compared to 18S rRNA qPCR, with borderline significance (McNemar’s Chi^2^, *p*_TARE-2_ = 0.068, *p*_*var*ATS_ = 0.083). Prevalence values were 25% (CI_95_ [21%–29%]) by LM, 50% (CI_95_ [45%–54%]) by 18S rRNA qPCR, and 58% (CI_95_ [53%–63%]) by *var*ATS qPCR or TARE-2 qPCR (Fig 2A). Applying ultra-sensitive techniques thus revealed a larger submicroscopic infection pool than detected by the routinely used molecular method. Despite a gain in prevalence of 25% over LM, 18S rRNA qPCR still underestimated the true parasite prevalence by 8% without major differences across age groups (Fig 2B). In a total of 295 *P*. *falciparum* infections, 16% (48 samples) were not detected by 18S rRNA qPCR but only by *var*ATS or TARE-2 qPCR (Fig 2C). Agreement between assays was very good in the subset positive in 18S rRNA qPCR, with all samples positive in 18S rRNA qPCR detected also by *var*ATS qPCR and all but two by TARE-2 qPCR. The level of agreement between TARE-2 and *var*ATS qPCRs in this sample subgroup was also high, with 79% (38/48) of samples detected by both ultra-sensitive assays.

**Fig 3:**
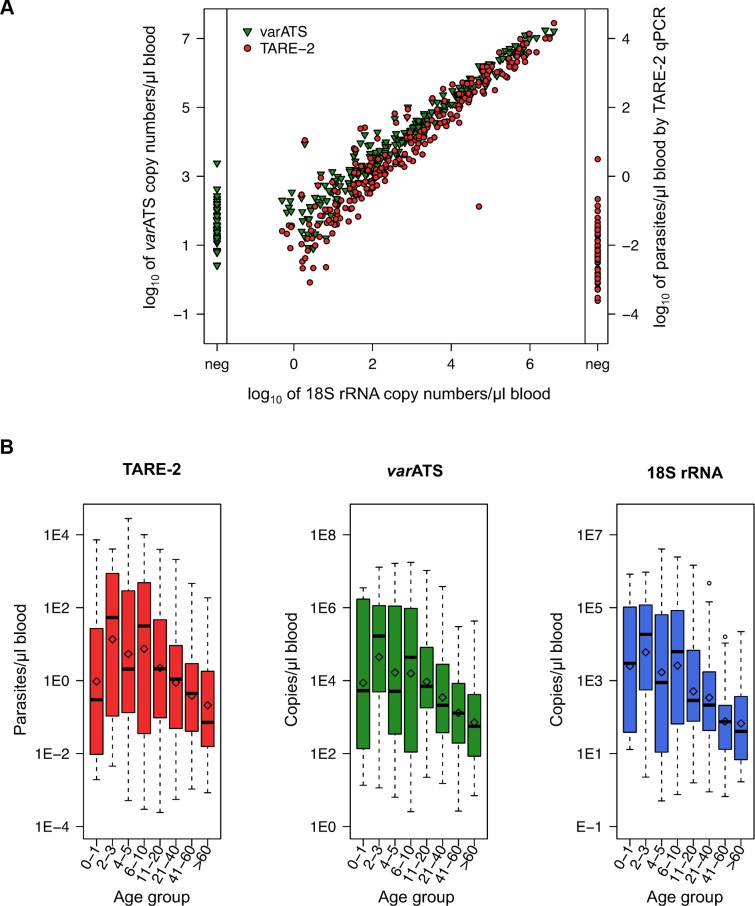
Correlation of parasite quantification using *var*ATS, TARE-2, and 18S rRNA qPCRs and parasite densities in Rufiji, Tanzania. (A) Parasite quantities determined by ultra-sensitive assays and their correlation with 18S rRNA quantification. Quantification was done relative to copy numbers of plasmid standards (18S rRNA, *var*ATS) or a parasite dilution row (TARE-2). Quantities of samples negative in 18S rRNA qPCR but positive in ultra-sensitive assays are shown in the left (*var*ATS) and right (TARE-2) panels. (B) *P*. *falciparum* densities based on TARE-2, *var*ATS, and 18S rRNA qPCRs by age (in years). The geometric mean in each age group is marked by a diamond; the median is denoted by a black line.

**Fig 4:**
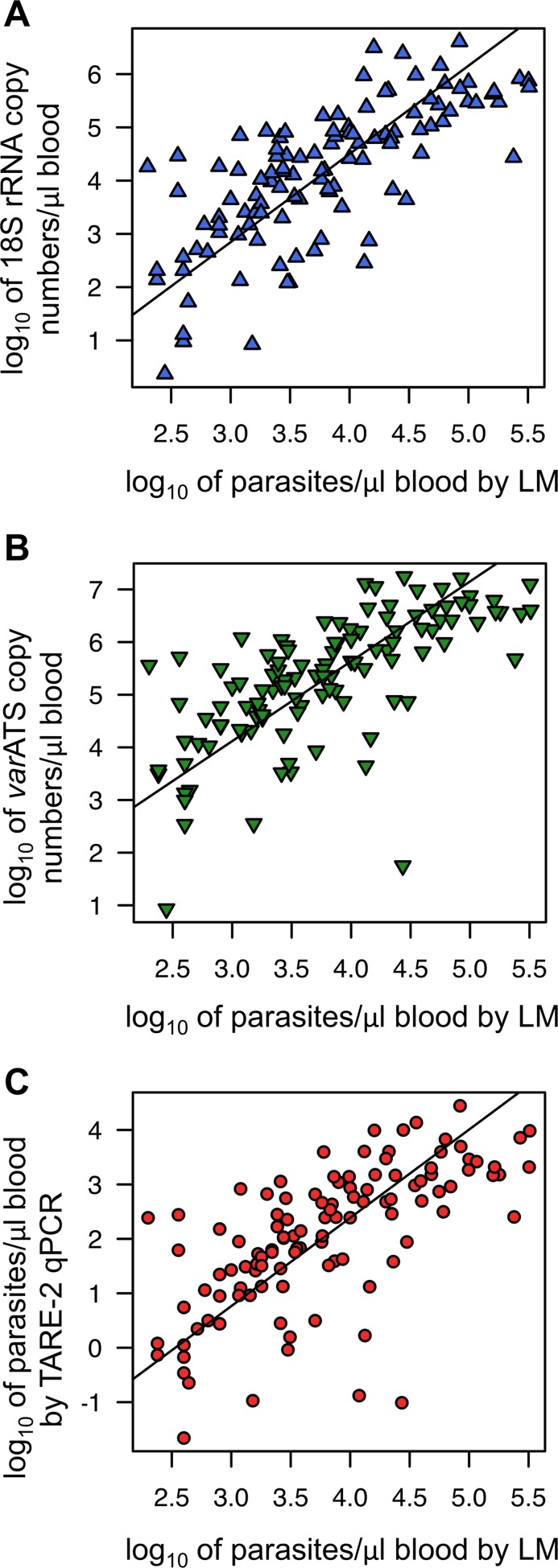
Correlation of parasite quantification by the three qPCR assays and light microscopy. Parasite quantities determined by 18S rRNA (A), *var*ATS (B), and TARE-2 (C) qPCRs and their correlation with parasite density by LM. Quantification by PCR was done relative to copy numbers of plasmid standards (18S rRNA, *var*ATS) or a parasite dilution row (TARE-2). For quantification by LM, 200 fields of a thick film were examined, and parasite density was calculated assuming 8,000 leucocytes/μl blood. Pearson’s product-moment correlation was used to assess correlation strength, and Deming regression was used to calculate regression lines.

Quantification of parasite load by *var*ATS and TARE-2 correlated very well with 18S rRNA qPCR quantification in field samples from Rufiji (Pearson’s correlation coefficient, *R* ^2^ = 0.98, CI_95_ [0.97–0.98], and *R* ^2^ = 0.95, CI_95_ [0.94–0.96], respectively; Fig 3A), as well as with each other (*R* ^2^ = 0.97, CI_95_ [0.96–0.98]). Correlation of parasite load determined by qPCR and by microscopy was similar for the three assays and ranged from 0.74 (18S rRNA, CI_95_ [0.64–0.81]) to 0.66 (TARE-2, CI_95_ [0.54–0.75]; *var*ATS: 0.68, CI_95_ [0.57–0.80]; Fig 4). Despite the high number of target sequences and slight sequence degeneration, quantification of parasite load is thus feasible using *var*ATS and TARE-2 qPCRs. Parasite loads by TARE-2 qPCR in samples negative by 18S rRNA qPCR were, except for few outliers, within the lowest quartile of all parasite loads by TARE-2 quantification. The same was observed for *var*ATS copy numbers of samples negative by 18S rRNA. When stratified by age, parasite densities or target copy numbers were low in infants up to 1 y, peaked in 2- to 3-y-old children, and decreased thereafter, with the lowest parasite loads observed in the oldest age group (Fig 3B).

The prevalence of gametocytes by *pfs25* qRT-PCR was 40% in all study participants (CI_95_ [36%–45%]). The proportion of *pfs25*-positive samples was highest in samples that were positive by LM, of which 77% (CI_95_ [69%–84%], 97/126) carried gametocytes (Fig 2D). Among submicroscopic infections identified by 18S rRNA qPCR, gametocytes were detected in 63% (CI_95_ [55%–72%], 85/134) of samples. In samples positive only by TARE-2 and/or *var*ATS qPCR, 40% (CI_95_ [26%–56%], 18/45) carried gametocytes. These observations prove that molecularly determined gametocyte carriers are not predominantly found among LM-positive individuals, but rather that an equal number of gametocyte carriers are present in study participants with submicroscopic infections. By use of a routinely used 18S rRNA assay, 16% of asexual infections and 9% of gametocyte carriers would have been missed.

### Performance on Sample Pools

To investigate the potential of our assays for a wider application in malaria surveillance or epidemiological field studies, we tested the power of all three qPCR assays to identify *P*. *falciparum*–positive samples in pools of five or ten samples, each containing one low-density *P*. *falciparum* infection. 18S rRNA qPCR failed to identify the two positive ten-sample pools and identified only one of five positive five-sample pools. In contrast, *var*ATS and TARE-2 qPCR correctly detected all positive five- and ten-sample pools. No amplification was observed from negative control pools. Our ultra-sensitive assays thus proved suitable for detection of low-grade infections after dilution in nine negative samples. These infections would be missed by 18S rRNA qPCR after pooling. In a setting with 2% *P*. *falciparum* prevalence, as simulated here, the cost of sample processing and detection can therefore be reduced by at least 70% without loss in sensitivity if ultra-sensitive assays are applied to pools of ten samples.

## Discussion

### Detecting Low-Density Infections Using Ultra-Sensitive Methods Is Relevant for Malaria Control Efforts

Accurate data on parasite prevalence in the community are imperative for targeting antimalarial interventions and for monitoring their outcome. In this study, we provide first evidence of very-low-grade infections in individuals who had previously been considered parasite-free, even after molecular diagnosis, and show that a large proportion of these samples carry gametocytes. In Rufiji, a high-endemic area in TZ, microscopic and submicroscopic infections (by standard 18S rRNA qPCR) each amount to roughly 40% of all *P*. *falciparum* infections; 16% are of ultra-low density and detected only by TARE-2 and *var*ATS assays. These ultra-low-density infections potentially contribute to transmission, as they represent 9% of the molecularly detected gametocyte carriers. In Maprik District, PNG, 18S rRNA qPCR failed to identify similar quantities of ultra-low-density infections.

A meta-analysis of infection prevalence across the endemicity spectrum has indicated that submicroscopic infections are generally more prevalent in low-transmission settings than in high-transmission areas [5], probably as a result of a recently reduced force of infection and the long duration of asymptomatic untreated infections [53–55]. In such areas, detection of infection, rather than assessment of malaria-associated illness, could serve as a better measure of the malaria burden and a better parameter for surveillance and evaluation [1,2]. Low-density infections can be missed in cross-sectional studies even when using standard 18S rRNA qPCR because parasitemia fluctuates and may occasionally fall below the detection threshold of the assay. Waves of asexual parasitemia and gametocytemia were described in malaria therapy data [56,57]. Accordingly, scanty infections may rise in density at a later time point and increase gametocyte production to detectable levels, leading to higher transmission potential. Improved measures of prevalence using tools for reliable detection of low-density infections can contribute significant information and are important for accurate monitoring and evaluation of malaria control activities, as well as for assessing the potential for onward transmission from human hosts to mosquitoes.

### Gametocyte Carriage in Low-Density Infections Emphasizes Their Potential Contribution to Malaria Transmission

Few studies have investigated the transmission potential of submicroscopic infections. Microscopically detectable infections with gametocyte densities below the microscopical threshold can infect mosquitoes, albeit at lower rates than microscopically gametocyte-positive samples (2.3% versus 13.2% infected mosquitoes) [58]. A meta-analysis of mosquito feeding assays conducted in several African countries showed that 27.6% of individuals who lacked microscopically detectable gametocytes were capable of infecting mosquitoes [59]. Similarly, data from the mid-20th century and from two recent studies showed that even blood from infections without any microscopically detectable parasites resulted in 0.2%–3.2% infected mosquitoes [5,60–62]. In a study performed in the Gambia, multiple parasite genotypes were detected in oocysts after feeding mosquitoes on blood seemingly carrying a clonal infection [63]. In that study, multiple gametocyte genotypes were detected in the same blood sample, suggesting that parasite clones undetectable on DNA level produced gametocytes in quantities sufficient to transmit to mosquitoes. Clustering of gametocytes, especially in infections with low gametocyte densities, has been given as a possible explanation for why such infections are able to transmit to mosquitoes [64–66]. A modeling analysis using data from Cameroon found that asexual densities did not predict the proportion of infected mosquitoes, contrary to gametocyte densities, which exhibited a complex and non-linear correlation with transmission success [58,67,68]. Taken together, the available data suggest that all infections should be viewed as potentially relevant for transmission. The relative contribution of low-density infections to forward transmission to mosquitoes hence may gain substantial importance in areas where these account for a large proportion of infections [5,58]. In our TZ setting, the majority of infections were submicroscopic and harbored 50% of gametocyte-positive samples. TARE-2 and *var*ATS assays identified a so far ignored extent of submicroscopic infective burden, with 40% of these low-key infections carrying gametocytes. We thus argue for including ultra-low-grade infections into the evaluation of malaria interventions and for acknowledging their potential relevance for maintaining transmission, a role that urgently needs experimental clarification.

### Sensitivity as a Major Determinant of Prevalence Estimates: Advantages and Limitations of the TARE-2 and *var*ATS qPCRs

Our results highlight the fact that prevalence data are strongly dependent on the sensitivity of the diagnostic technique applied. Even if parasite prevalence is measured using standard qPCR protocols, many low-key infections remain undetected. Standard PCR is widely considered a molecular gold standard of malaria diagnosis complementing LM, the traditional gold standard, yet our results suggest that this notion requires revision. It becomes increasingly clear that the volume of blood analyzed [44] and the use of multi-copy markers to increase the representation of a PCR template in the amplification reaction ([19] and this study) have great influence on the prevalence outcome. Our findings shed new light on MSAT strategies for interruption of transmission in elimination settings, particularly those that rely on RDT-based diagnosis only, as it becomes clear that the ignored proportion of submicroscopic infections is even larger than anticipated. Following a recent MSAT campaign in TZ, RDT-undetected infections were given as a plausible explanation for the short-lived effect on malaria episode incidence [69]. In that study, it was estimated that more than 45% of PCR-detectable infections were missed by RDT, which, given our results, is very likely a substantial underestimation. A major task now consists in adapting molecular methods with enhanced sensitivity to meet the requirements of a robust, field-compatible diagnostic assay. Such tools are becoming increasingly important to determine the infection burden irrespective of endemicity level.

We have presented here two ultra-sensitive qPCR assays for improved detection of low-grade *P*. *falciparum* infections and their application to sample pools. The *var*ATS qPCR is very robust and highly specific, and allows fast and easy data analysis through the use of a sequence-specific probe. The TARE-2 assay is more susceptible to changes in the chemical composition of the DNA solution and requires melt curve analysis of amplicons, which is a potential drawback, particularly when performed by less-trained personnel. Both assays exhibited slightly suboptimal amplification efficiency despite all optimization efforts, possibly because of a wobble base introduced into primer and probe sequences to improve annealing to the target copies in the genome. Regardless of this inherent low efficiency, sensitivity was superior to that of 18S rRNA qPCR in field samples and on parasite dilution rows. Surprisingly, the TARE-2 qPCR did not outperform the *var*ATS assay despite substantially higher target numbers in the genome. This might be explained by the degenerate sequence of the TARE-2 repeat units or by the clustered distribution of the repeats at chromosome ends. In the *3D7* genome, about ten TARE-2 tandem repeats are present at 24 chromosome ends, and in this arrangement, they may not be separated during DNA extraction. The 59 *var*ATS targets of strain *3D7* also localize to chromosome ends and a few intracellular loci. We assume an equal probability for both targets to be represented in a PCR reaction, but certainly both assays surpass 18S rRNA qPCR, with three copies on different chromosomes targeted by our assay. Because of the need for advanced laboratory infrastructure and staff training, use of our TARE-2 and *var*ATS qPCRs in their current setup is not feasible in remote field settings. However, the assays are ideally suited for use in reference laboratories, for example for quality assurance or for centralized processing of large sample numbers in sample pools. Several strategies for pooling samples for malaria surveys have been described, comprising one or several pooling steps before [70–72] or after [73,74] DNA extraction. Pooling is severely limited by its inherent diluting effect and is therefore not recommended in the *Malaria* Eradication Research Agenda (malERA) strategy [1]. In low-endemic settings, in particular, where pooling would be most cost- and labor-effective, submicroscopic infections are highly prevalent [5] but are most likely missed in pools because of their low densities. Our *var*ATS and TARE-2 assays proved to be useful for testing pooled samples as they counterbalance the diluting effect through multiple marker copies per parasite. In our hands, even the lowest-density infections diluted with nine negative samples were still detectable. This high sensitivity may be further enhanced by increasing the volume of blood samples and concentrating material before qPCR [44]. The availability of ultra-sensitive assays such as our TARE-2 and *var*ATS qPCRs makes sample pooling without loss in sensitivity feasible and allows achieving higher throughput in the context of limited resources in large-scale field studies. Once similar assays have been developed also for detecting the other human-infecting *Plasmodia*, blood pooling followed by multiplex PCR will further reduce the per-sample cost in studies requiring detection of all four *Plasmodium* species.

### Conclusion

In conclusion, we encourage employing assays with enhanced sensitivity, such as the TARE-2 or *var*ATS qPCRs, in any malaria survey aiming to obtain accurate prevalence data and for monitoring intervention success, and recommend them particularly for screening of community samples in areas of low endemicity. The fact that parasites are more prevalent than currently thought has consequences for malaria control efforts, some of which are based on identifying all infected individuals, and this fact must be acknowledged by all users of prevalence data such as health officials, strategy planners, and mathematical modelers. Infections of ultra-low densities in our TZ samples carried gametocytes in 40% of cases, and thus it is highly probable that they can be transmitted to mosquitoes at the time point of the survey or later. Until the infectiousness to mosquitoes of low-density infections has been clarified, applying the most sensitive tools is essential for better defining the true infection burden and informing elimination strategies.

## Supporting Information

S1 Fig Melting temperature of TARE-2 amplicons using DNA samples from two different sources (surveys in Tanzania and Papua New Guinea). Melting temperature (*T*_m_) of true positives (as in positive control/standards) differs significantly from false positive signals (primer dimer, Welch’s *t*-test, p < 0.001). Owing to the degenerate character of the TARE-2 repeat unit, PCR products vary in sequence composition, which is reflected in slight variations in the *T*_m_ of true positives (TZ, 68.6–72.2°C; PNG, 70.0–72.1°C). Different DNA extraction kits and dilution buffers used in the PNG and TZ surveys cause shifts in *T*_m_ for both specific amplicons and primer dimer. The mean *T*_m_ of true positives and primer dimer was significantly different between the PNG and TZ samples (Welch’s *t*-test, *p* < 0.001), while qPCR amplicons amplified from *3D7* DNA standard included on both the TZ and PNG qPCR plates showed no significant differences in their mean *T*_m_. The *T*_m_ of specific amplicons and primer dimer was hence established separately for each of our two sets of field samples.
(TIFF)

S1 Table Oligonucleotide sequences and qPCR conditions for *var*ATS and TARE-2 assays. Primers were purchased from Eurofins. The *var*ATS probe and all qPCR reagents were purchased from Applied Biosystems/Life Technologies.
(DOCX)

S2 Table STARD checklist for reporting studies of diagnostic accuracy. (DOCX)

S3 Table Results of TARE-2, *var*ATS, and 18S rRNA qPCRs on parasite dilution rows. (DOCX)

S4 Table Database of light microscopy and qPCR results of the Tanzanian cross-sectional study. Age_Group: in years, corresponding to Figs 2 and 3; 18SrRNA_Quantification: 18S rRNA copy numbers per microliter of blood; *var*ATS_Quantification: *var*ATS copy numbers per microliter of blood; TARE-2_Quantification: parasites per microliter of blood as determined by TARE-2 qPCR; LM_Quantification: parasites per microliter of blood as determined by LM; Pfs25_positivity: 1 indicates positive in *pfs25* qRT-PCR, 0 indicates negative in *pfs25* qRT-PCR.
(XLSX)

S1 Text Generation of *P*. *falciparum* dilution rows for determining the limit of detection and qPCR efficiency. (DOCX)

## Abbreviations:

18S rRNA: 18S small subunit ribosomal RNA
gDNA: genomic DNA
LAMP: loop-mediated isothermal amplification
LM: light microscopy
LOD: limit of detection
MDA: mass drug administration
MSAT: mass screening and treatment
NASBA: nucleic acid sequence-based amplification
*Pf*EMP1: *P*. *falciparum* erythrocyte membrane protein 1
PNG: Papua New Guinea
qPCR: quantitative PCR
qRT-PCR: quantitative reverse-transcription PCR
RDT: rapid diagnostic test
RPA: isothermal recombinase polymerase amplification
TARE-2: telomere-associated repetitive element 2
TZ: Tanzania
*var*ATS: *var* gene acidic terminal sequence
