# Supplementary figures and images for: Ultra-Sensitive Detection of Plasmodium falciparum by Amplification of Multi-Copy Subtelomeric Targets
Source: PLoS Med. 2015 Mar 3;12(3):e1001788. doi: 10.1371/journal.pmed.1001788 (PMC4348198; doi:10.1371/journal.pmed.1001788)

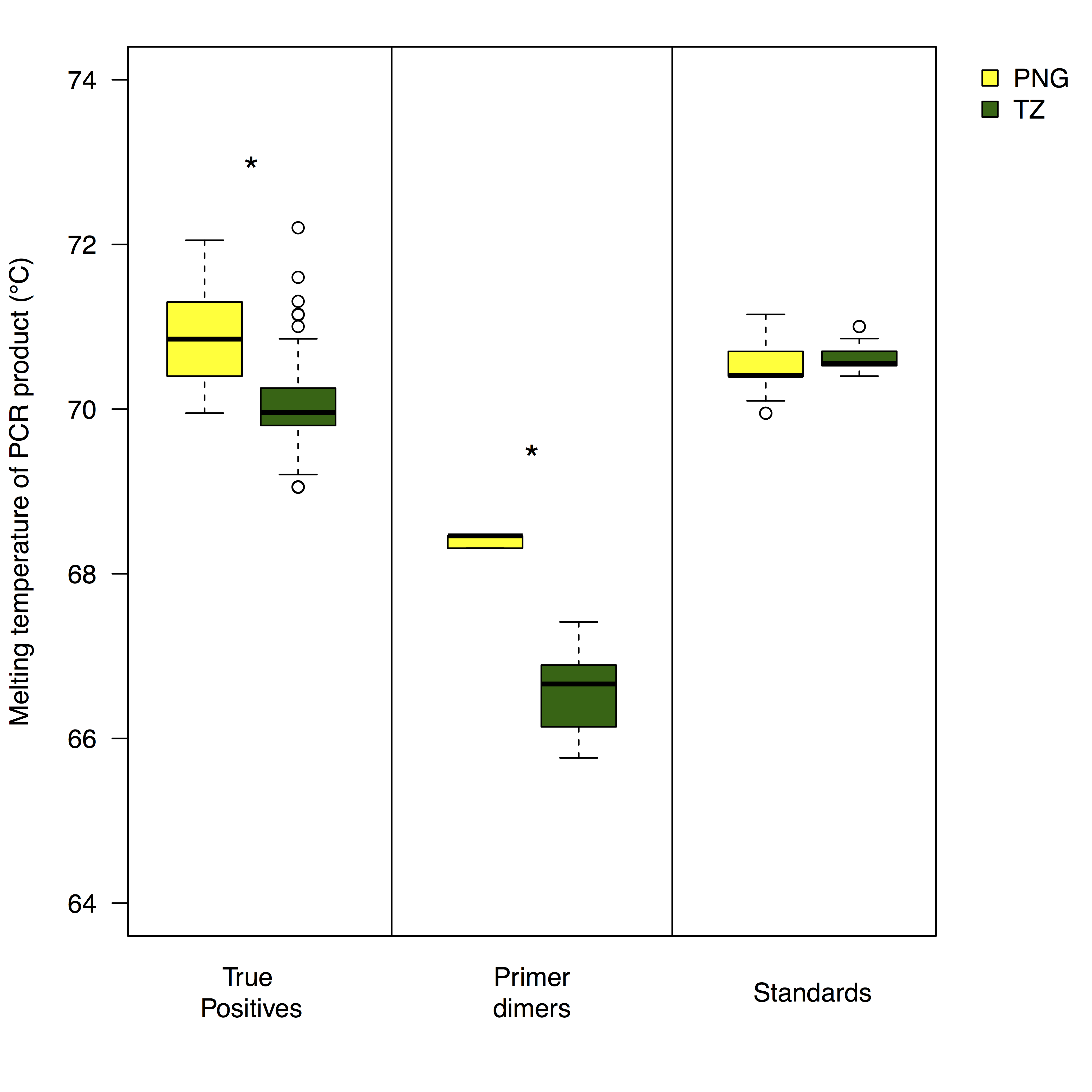

Supplement: S1 Fig — Melting temperature (T m) of true positives (as in positive control/standards) differs significantly from false positive signals (primer dimer, Welch’s t-test, p < 0.001). Owing to the degenerate character of the TARE-2 repeat unit, PCR products vary in sequence composition, which is reflected in slight variations in the T m of true positives (TZ, 68.6–72.2°C; PNG, 70.0–72.1°C). Different DNA extraction kits and dilution buffers used in the PNG and TZ surveys cause shifts in T m for both specific amplicons and primer dimer. The mean T m of true positives and primer dimer was significantly different between the PNG and TZ samples (Welch’s t-test, p < 0.001), while qPCR amplicons amplified from 3D7 DNA standard included on both the TZ and PNG qPCR plates showed no significant differences in their mean T m. The T m of specific amplicons and primer dimer was hence established separately for each of our two sets of field samples. (TIFF) [file pmed.1001788.s001.tiff]
