# Supplementary material for: Ultra-Sensitive Detection of Plasmodium falciparum by Amplification of Multi-Copy Subtelomeric Targets
Source: PLoS Med. 2015 Mar 3;12(3):e1001788. doi: 10.1371/journal.pmed.1001788 (PMC4348198; doi:10.1371/journal.pmed.1001788)
Supplement: S1 Table — Primers were purchased from Eurofins. The varATS probe and all qPCR reagents were purchased from Applied Biosystems/Life Technologies. (DOCX) [file pmed.1001788.s002.docx]

**S1 Table. Oligonucleotide sequences and qPCR conditions for *var*ATS and TARE-2 assays.** Primers were purchased from Eurofins. The *var*ATS probe and all qPCR reagents were purchased from Applied Biosystems/Life Technologies.

|  | varATS | TARE-2 |
| --- | --- | --- |
| Oligonucleotide sequences | | |
| Primer-fw (5’-3’) | cccatacacaaccaaytgga | ctatgttgcacttacatgcayaat |
| Primer-rev (5’-3’) | ttcgcacatatctctatgtctatct | tgacctaagaagtavaataatgatga |
| Probe (5’-3’) | 6-FAM-trttccataaatggt-NFQ-MGB | - |
| qPCR reaction conditions (final concentration in qPCR mix) | | |
| Total volume | 12 (25)^$^ | 25 (25)^$^ |
| DNA volume | 4 (5)^$^ | 4 (5)^$^ |
| TaqMan^®^ Gene Expression Mastermix | 1x | - |
| Power SYBR^®^ Green mix | - | 1x |
| Primer (each fw & rev) | 800 nM | 200 nM |
| Probe | 400 nM | - |
| qPCR cycling conditions | | |
| Pre-incubation | 2 min – 50°C | 2 min – 50°C |
| Initial denaturation | 10 min – 95°C | 10 min – 95°C |
| Denaturation | 15 sec – 95 °C | 15 sec – 95 °C |
| Annealing & Elongation | 1 min – 55°C | 1 min – 57°C |
| Number of cycles | 45 | 45 |
| Melt Curve | - | 57-95°C, 0.3°C increment |
| Positivity threshold | 0.07 | 0.07 |
| Standard material for quantification | Plasmid | gDNA of parasite dilution row |
| Platform | StepOne Plus^®^ Real-Time PCR System (Applied Biosystems) | StepOne Plus^®^ Real-Time PCR System (Applied Biosystems) |

^$^ Brackets: volumes used for sensitivity and specificity tests on parasite culture and for PNG samples.
