# Supplementary material for: Ultra-Sensitive Detection of Plasmodium falciparum by Amplification of Multi-Copy Subtelomeric Targets
Source: PLoS Med. 2015 Mar 3;12(3):e1001788. doi: 10.1371/journal.pmed.1001788 (PMC4348198; doi:10.1371/journal.pmed.1001788)
Supplement: S3 Table — (DOCX) [file pmed.1001788.s004.docx]

**S3 Table. Results of TARE-2, *var*ATS, and 18S rRNA qPCRs on parasite dilution rows.**

A) Serial dilution of ring-stage parasite *in vitro* culture (*3D7* strain)

|  | 18S rRNA | | *var*ATS | | TARE-2 | |
| --- | --- | --- | --- | --- | --- | --- |
| Parasites/µl blood | Positivity | Mean C_t_ (±StDev) | Positivity | Mean C_t_ (±StDev) | Positivity | Mean C_t_ (±StDev) |
| 6800 | 3/3 | 26.3 ± 0.5 | 3/3 | 19.9 ± 0.3 | 3/3 | 16.9 ± 1.0 |
| 680 | 3/3 | 30.4 ± 0.5 | 3/3 | 23.8 ± 0.4 | 3/3 | 21.1 ± 1.4 |
| 68 | 3/3 | 34.1 ± 0.5 | 3/3 | 27.5 ± 0.2 | 3/3 | 24.7 ± 1.7 |
| 6.8 | 3/3 | 37.6 ± 0.7 | 3/3 | 30.8 ± 0.1 | 3/3 | 28.6 ± 1.6 |
| 3.4 | 3/3 | 38.7 ± 0.3 | 6/6 | 32.0 ± 0.2 | 6/6 | 29.7 ± 1.4 |
| 0.68 | 3/3 | 40.8 ± 1.2 | 6/6 | 34.4 ± 0.3 | 6/6 | 31.9 ± 1.3 |
| 0.34 | 3/3 | 42.7 ± 0.8 | 6/6 | 35.5 ± 0.2 | 6/6 | 32.6 ± 1.7 |
| 0.068 | 1/3 | - | 6/6 | 37.3 ± 0.3 | 6/6 | 38.5 ± 2.0 |
| 0.034 | 0/3 | - | 6/6 | 37.7 ± 0.5 | 5/6 | 42.0 ± 1.5 |
| 0.0068 | 0/3 | - | 1/6 | - | 0/6 | - |
| 0.0034 | 2/3 | - | 2/6 | - | 2/6 | - |
| 0.00068 | 0/3 | - | 1/6 | - | 1/6 | - |

B) Serial dilution of the WHO international standard for *P. falciparum* DNA for nucleic amplification techniques

|  | *var*ATS | | TARE-2 | |
| --- | --- | --- | --- | --- |
| Parasites/µl blood | Positivity | Mean C_t_ (±StDev) | Positivity | Mean C_t_ (±StDev) |
| 47000 | 3/3 | 18.7 ± 0.1 | 3/3 | 18.9 ± 0.3 |
| 4700 | 3/3 | 22.9 ± 0.1 | 3/3 | 23.0 ± 0.8 |
| 470 | 3/3 | 26.5 ± 0.1 | 3/3 | 27.4 ± 0.5 |
| 47 | 3/3 | 30.1 ± 0.3 | 3/3 | 31.5 ± 0.8 |
| 23.5 | 5/5 | 30.8 ± 0.2 | 5/5 | 33.3 ± 0.6 |
| 4.7 | 5/5 | 33.6 ± 0.2 | 5/5 | 36.3 ± 1.0 |
| 2.3 | 5/5 | 34.5 ± 0.2 | 5/5 | 37.9 ± 0.7 |
| 0.47 | 5/5 | 36.8 ± 0.4 | 5/5 | 41.2 ± 1.6 |
| 0.23 | 5/5 | 38.0 ± 1.0 | 5/5 | 40.9 ± 1.3 |
| 0.047 | 4/5 | 39.9 ± 1.1 | 5/5 | 41.6 ± 0.6 |
| 0.023 | 2/5 | - | 2/5 | - |
| 0.0047 | 2/5 | - | 0/5 | - |
| 0.0023 | 0/5 | - | 0/5 | - |
