# Supplementary material for: Ultra-Sensitive Detection of Plasmodium falciparum by Amplification of Multi-Copy Subtelomeric Targets
Source: PLoS Med. 2015 Mar 3;12(3):e1001788. doi: 10.1371/journal.pmed.1001788 (PMC4348198; doi:10.1371/journal.pmed.1001788)
Supplement: S1 Text — (DOCX) [file pmed.1001788.s006.docx]

**S1 Text: Generation of *P. falciparum* dilution rows for determining the limit of detection and qPCR efficiency.**

Parasite *in vitro* culture (*3D7* strain):

After 2 rounds of synchronization (5% sorbitol), ring-stage parasites were quantified by microscopic examination of 15 fields of a Giemsa-stained thin film by counting parasites versus red blood cells (RBC). In total >2000 RBC were screened. Parasite concentration in the blood pellet was calculated based on RBC counts of two representative blood pellets in a Neubauer-counting chamber. A serial dilution of parasite culture was produced in PBS and diluted 1:10 in whole blood from a malaria-negative blood donor. For each dilution, DNA was isolated in triplicate from 100 µl blood using the QIAamp 96 DNA Blood Kit (Qiagen), eluted in 100 µl TE-buffer and stored at -20°C. For determination of assay sensitivity, each assay was performed on the extracted triplicates of all dilutions, once for high-density dilutions (6.8x10^4^ to 6.8 parasites/µl) and twice for low-density dilutions (3.4 to 0.00068 parasites/µl).

WHO international standard for *P. falciparum* DNA for nucleic amplification techniques:

The lyophilized material was diluted in 0.5 ml sterile water according to NIBSC protocol. After reconstitution, parasite density in the sample was 4.7x10^5^ parasites per µl [27, 52]. A serial dilution was produced in whole blood from a malaria-negative blood donor. For each dilution, DNA was isolated using the QIAamp 96 DNA Blood Kit (Qiagen) in triplicate (4.7x10^5^ to 47 parasites/µl) or in quintuple (23.5 to 4.7x10^-3^ parasites/µl) from 200 µl blood and eluted in 200 µl TE-buffer. qPCR was performed on the extraction triplicates or quintuples.
